# Fatigue is associated with disease activity in some, but not all, patients living with rheumatoid arthritis: disentangling “between-person” and “within-person” associations

**DOI:** 10.1186/s41927-021-00230-2

**Published:** 2022-01-07

**Authors:** Grada A. Versteeg, Peter M. ten Klooster, Mart A. F. J. van de Laar

**Affiliations:** Department of Psychology, Health and Technology, Faculty of Behavioural, Management and Social Sciences, University of Twente, P.O. box. 217, 7500 AE Enschede, The Netherlands

**Keywords:** Rheumatoid arthritis, Disease activity, Fatigue, Within-patients associations, Between-patients associations

## Abstract

**Background:**

Previous research has shown an unclear and inconsistent association between fatigue and disease activity in patients with rheumatoid arthritis (RA). The aim of this study was to explore differences in “between-person” and “within-person” associations between disease activity parameters and fatigue severity in patients with established RA.

**Methods:**

Baseline and 3-monthly follow-up data up to one-year were used from 531 patients with established RA randomized to stopping (versus continuing) tumor necrosis factor inhibitor treatment enrolled in a large pragmatic trial. Between- and within-patient associations between different indicators of disease activity (C-reactive protein [CRP], erythrocyte sedimentation rate [ESR], swollen and tender joint count [ SJC and TJC], visual analog scale general health [VAS-GH]) and patient-reported fatigue severity (Bristol RA Fatigue Numerical Rating Scale) were disaggregated and estimated using person-mean centering in combination with repeated measures linear mixed modelling.

**Results:**

Overall, different indices of disease activity were weakly to moderately associated with fatigue severity over time (β’s from 0.121 for SJC to 0.352 for VAS-GH, all *p*’s < 0.0001). Objective markers of inflammation (CRP, ESR and SJC) were associated weakly with fatigue within patients over time (β’s: 0.104–0.142, *p*’s < 0.0001), but not between patients. The subjective TJC and VAS-GH were significantly associated with fatigue both within and between patients, but with substantially stronger associations at the between-patient level (β’s: 0.217–0.515, *p*’s < 0.0001). Within-person associations varied widely for individual patients for all components of disease activity.

**Conclusion:**

Associations between fatigue and disease activity vary largely for different patients and the pattern of between-person versus within-person associations appears different for objective versus subjective components of disease activity. The current findings explain the inconsistent results of previous research, illustrates the relevance of statistically distinguishing between different types of association in research on the relation between disease activity and fatigue and additionally suggest a need for a more personalized approach to fatigue in RA patients.

*Trial registration* Netherlands trial register, Number NTR3112.

## Background

Fatigue, although prioritized by patients, is a poorly understood symptom of rheumatoid arthritis (RA). Fatigue is reported by almost 90% of patients with RA and around 40% of patients report clinically important levels of fatigue or severe fatigue [1, 2]. Qualitative research among RA patients with severe fatigue suggests that their fatigue is different from normal tiredness and is perceived by them as having far reaching consequences for all domains of daily life, by being intrusive and overwhelming [3]. Many patients prioritize fatigue as an important health outcome [4, 5] and therefore fatigue is now a core outcome measure in RA studies [6, 7]. Rheumatologists acknowledge the prevalence and impact of fatigue in their RA patients as 93% of the respondents of a questionnaire sent to rheumatologisst and trainees indicate that fatigue should still be considered a problem for patients even if pain is successfully resolved [8]. Despite this, fatigue is rarely a treatment target. This is mainly due to the lack of knowledge about the (patho)physiology of fatigue and the role of RA, in particular disease activity [9]. Although RA patients generally mention their disease as the cause of their fatigue [10], for markers of inflammation and other indicators of disease activity, an unclear and inconsistent relationship with fatigue in RA has been shown. Some studies, showing a significant relation, contrast other studies in which inflammatory markers did not contribute to the severity of fatigue at all [11, 12]. Although treatment with biologicals comes with reduction of fatigue in RA [9, 13], the actual group-level effects of biologicals on fatigue appears to be small [14]. Moreover, the reduction of fatigue is driven by improvements in pain and depression, and not by changes in inflammatory activity [15].

Our current understanding of the relation between fatigue in RA and disease activity is limited by the fact that, up to present, studies have been either small and cross-sectional or longitudinal with only a limited number of observations per patient [12]. Importantly, cross-sectional studies are by design only able to examine so-called “*between-person”* associations. As such, these studies only examined if patients with higher disease activity than others also experienced more fatigue. Listening to patients and their physicians, that fatigue and its relationship with RA differs between persons, demands for methodologies that can distinguish “between-person” from “within-person” associations. Such “within-person*”* relations over time describe what happens with fatigue within individual patients when their disease activity changes.

Most theories about the mechanisms that underlie specific associations between variables of interest, such as disease activity and fatigue, but also interventions targeting such variables, are based on processes that are assumed to take place within persons [16, 17]. So, they assume that changing one variable leads to changes in the other variable within people. Results from “between-person” analysis, such as those from cross-sectional studies, can only be generalized to “within-person” relations when strict assumptions of statistical “ergodicity” are met [18, 19]. However, it has been convincingly demonstrated that these assumptions are rarely met, and that “between-person” relations can be quite different from “within-person” relations both in magnitude and sometimes even in direction [16–20]: an observation referred to as Simpson’s paradox [21]. For instance, our study examining the association between disease activity and radiographic progression in RA patients showed that different indices of disease activity were not or only weakly associated at the “between-patient” level, but more often and more strongly associated within individual patients over time [22].

Cross-sectional or even standard longitudinal analyses do not allow for examining within-person associations, since the latter also mix both between-person and within-person results. Instead, specific statistical analysis methods of longitudinal data are needed that can distinguish the multiple sources of information, such as multilevel (hierarchical) mixed models [16, 17, 19, 20, 23, 24]. Although disaggregating “within-person” and “between-person” effects is increasingly used in other fields, it has been scarcely used in medicine and up to present not for studying the relationship between fatigue and disease activity in RA. More detailed knowledge on the type of association between different indicators of disease activity and fatigue may shed light on the apparent inconsistent relationships found in previous studies and is mandatory to develop a theoretical framework for fatigue in RA [12]. If between-person associations are indeed different from within-person associations, this can also have relevant implications for improving treatment of RA fatigue as it may allow future identification of individual patients or groups of patients with different patterns of associations between disease activity and fatigue.

Therefore, the aim of this study was to explore the “between-person” and “within-person” association of disease activity indices and fatigue severity in patients with established stable RA, who were asked to withdraw their treatment with a tumor necrosis factor inhibitor (TNFi).

## Methods

### Patients and study design

Data were used from the Potential Optimalisation of Expediency and Effectiveness of TNFi’s (POET trial), registered in the Netherlands Trial Register (NTR3112) [25, 26]. Ethical approval for this multicenter study was granted by the Committee on Research Involving Human Subjects, region Arnhem—Nijmegen (Commissie Mensgebonden Onderzoek, regio Arnhem—Nijmegen). Local feasibility was approved by the Ethical Committees of all participating hospitals. In this pragmatic open-label trial, adult patients with established RA and stable low disease activity (28-joint Disease Activity Score [DAS28] < 3.2) for at least 6 months were randomized in a 2:1 ratio to stop or continue treatment with their current TNFi and followed up for one year. Concomitant treatment with conventional synthetic disease-modifying antirheumatic drugs was continued. In case of flare (DAS28 ≥ 3.2 with an increase > 0.6) [27], TNFi could be restarted at the discretion of the rheumatologist. In total, 817 patients were included in the POET trial. All analyses in the current study were performed using the data from the discontinuation group (N = 531) since this treatment arm contained the most patients and more changes in both disease activity and fatigue was observed in this group over time.

### Assessments

Patients’ disease activity was evaluated at the outpatient clinic by their treating rheumatologist and rheumatology nurse at baseline and at least every three months thereafter, for a period of one year. Additionally, patients completed patient-reported outcome measurements every three months, including fatigue.

#### Disease activity

Disease activity measurements included the erythrocyte sedimentation rate (ESR, mm/hour), C-reactive protein level (CRP, mg/dl), 28 tender and swollen joint counts (TJC and SJC), and a patient-reported assessment of general health on a 100-mm visual analog scale (VAS-GH). ESR, joint counts and the VAS-GH were used to calculate the composite DAS28-ESR [28].

#### Fatigue severity

The Bristol RA Fatigue (BRAF) scales [29, 30] were used to measure different dimensions of fatigue at the defined timepoints. For compatibility with previous studies, that most used single-item scales for fatigue severity or intensity, the numerical rating scale for fatigue severity (BRAF-NRS Severity) was used for all analyses. The BRAF-NRS Severity measures the average level of fatigue during the past 7 days on an 0–10 NRS anchored by “no fatigue” (0) to “totally exhausted” [10]. The BRAF-NRS Severity scale has demonstrated both strong reliability and adequate sensitivity to change [31]. The patient acceptable symptom state for 0–10 fatigue NRSs has been estimated to be around 4 on average in different RA populations [32, 33].

### Data analysis

All statistical analyses were performed using IBM SPSS Statistics version 26. Means of disease activity and fatigue scores were estimated and analysed using repeated measures linear mixed model analyses with time as a fixed covariate. Person-mean centering in combination with multilevel mixed modeling was used to separate within-patient associations between disease activity and fatigue from between-patient associations [17, 19, 20, 24]. For this, disease activity scores at each time-point were within-subject centered by subtracting the patient’s mean disease activity score across all available time-points from each observed value from that patient at the different time points. Within-subject centering effectively eliminates all between-subject variance, thus allowing to distinguish within-person effects from between-person effects in longitudinal models [17].

In the first series of models, observed disease activity values were entered as fixed time-varying covariates in repeated-measures linear mixed models with fatigue severity at each time point as dependent variable and patient intercept as random effect. In these models, the resulting regression estimate represents an aggregation (or unknow “blend”) of both the between-person and within-person effects of time-varying disease activity values on fatigue [17]. In the next series of models, person-mean disease activity (for between-person association) across all observations and time-varying person-mean centered disease activity at each observation (for within-person association) were simultaneously entered as fixed covariates. This procedure statistically separates any between-person association from within-person associations. The resulting person-mean regression estimate for disease activity indicates the extent to which patients’ mean disease activity scores are associated with fatigue (i.e., do patients with on average high disease activity report more severe fatigue at the different time points?). In contrast, the person-mean centered regression estimate indicates the extent to which patients’ deviations from their average (or typical) disease activity are associated with more or less fatigue at that that time point.

In all models person-mean centered disease activity scores, person-mean disease activity scores and fatigue severity scores were additionally converted to Z-scores to obtain standardized regression estimates (β) alongside unstandardized estimates from the mixed models. Standardized estimates were interpreted with Cohen’s [34] guidelines for small, medium, and large effect sizes (0.10, 0.30, and 0.50, respectively). Separate mixed models were estimated for composite DAS28-ESR scores and each of the individual disease activity parameters (ESR, CRP, TJC, SJC and VAS-GH) as fixed covariates. All models were estimated using the restricted maximum likelihood method and a compound symmetry covariance structure was used for the repeated measurements as this best fitted the data based on log-likelihood ratio tests across the different models. Between-patient and within-patient associations were illustrated using the ggplots2 package in R.

## Results

### Patient characteristics

Table 1 presents the baseline characteristics of the patients included in the TNFi discontinuation group. Patients were mostly female with a mean age of 60 years and on average longstanding disease. Baseline disease activity was low (DAS28 < 3.2), and the majority of patients reported a BRAF-NRS fatigue severity score < 4.

**Table 1:** Baseline characteristics of the patients in the discontinuation group

Characteristic	N = 531
Female sex, n (%)	362 (68.2)
Age, mean (SD) years	60.0 (11.8)
Disease duration, mean (SD) years	12.0 (8.8)
RF positive, n (%)	328 (67.5)
Anti-CCP positive, n (%)	332 (68.3)
Erosive disease, n (%)	305 (62.8)
DAS28-ESR, mean (SD)	1.98 (0.76)
ESR, median (IQR)	0 (5–18)
CRP, median (IQR)	2 (1–5)
TJC, median (IQR)	0 (0–0)
SJC, median (IQR)	0 (0–0)
VAS-GH	18.9 (19.2)
BRAF-NRS Severity, mean (SD)	3.7 (2.4)

*RF* rheumatoid factor; *anti-CCP* anti-cyclic citrullinated peptide; *DAS28-ESR* disease activity score in 28 joints including the erythrocyte sedimentation rate; *ESR* erythrocyte sedimentation rate; *CRP* C-reactive protein; *TJC* tender joint count; *SJC* swollen joint count; *VAS-GH* patient-reported assessment of General Health on a 100-mm visual analog scale; *BRAF-NRS* Bristol Rheumatoid Arthritis Fatigue-numeric rating scale

**Fig. 1:**
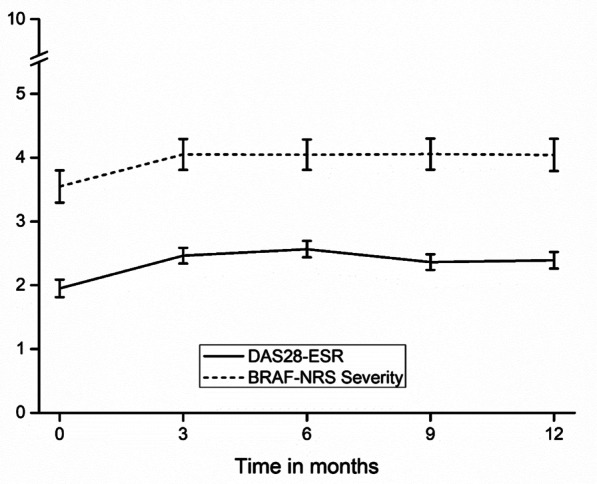
Estimated marginal means of disease activity and fatigue scores over time. DAS28-*ESR* disease activity score in 28 joints including the erythrocyte sedimentationrate; *BRAF-NRS* Bristol Rheumatoid Arthritis Fatigue-numeric rating scale; Error bars art 95% confidence intervals

Both disease activity scores (including all individual DAS-28 components) and fatigue scores significantly increased (all time effect *p*’s < 0.001) after stopping TNFi (Fig. 1). In total, 51.2% of the patients experienced a disease activity flare and almost half of the patients (47.5%) restarted their TNFi within 12 months after discontinuation.

### Aggregate associations between disease activity and fatigue

Composite DAS28-ESR scores were significantly, but only weakly, associated over time in 'aggregated' (or blended) between- and within-patient analysis (β = 0.274, *p* < 0.0001). Aggregate associations for the separate parameters of disease activity were also weak for CRP and ESR values and the SJC and TJC, but medium for the VAS-GH (Table 2).

**Table 2:** Overall (aggregate) associations between time-varying indices of disease activity and fatigue severity over time

	Estimate (95% CI)	Standardized estimate (95% CI)	t	*p*
DAS28-ESR	0.651 (0.508–0.795)	0.274 (0.214–0.334)	8.934	< 0.0001
CRP	0.041 (0.029–0.052)	0.150 (0.108–0.193)	6.922	< 0.0001
ESR	0.037 (0.027–0.046)	0.197 (0.146–0.247)	7.648	< 0.0001
SJC	0.192 (0.132–0.251)	0.121 (0.083–0.159)	6.315	< 0.0001
TJC	0.200 (0.158–0.243)	0.180 (0.141–0.218)	9.197	< 0.0001
VAS-GH	0.041 (0.035–0.046)	0.352 (0.304–0.400)	14.394	< 0.0001

*DAS28-ESR* disease activity score in 28 joints including the erythrocyte sedimentation rate; *CRP* C-reactive protein; *ESR* erythrocyte sedimentation rate; *SJC* swollen joint count; *TJC* tender joint count; *VAS-GH* patient-reported assessment of General Health on a 100-mm visual analog scale

### Disaggregated between-patient and within-patient associations

Disaggregated analysis showed that at the group level DAS28-ESR scores were significantly associated both between patients and within patients over time (Table 3). This indicates that patients with on average higher disease activity than other patients, also report more fatigue than other patients. In addition to this between-person effect, within individual patients increases in disease activity from their own mean were also associated with more severe fatigue.

**Table 3:** Disaggregated between-patient and within-patient associations between indices of disease activity and fatigue severity

	B (95% CI)	Standardized β (95% CI)	t	*p*
DAS28-ESR				
BP	0.732 (0.484–0.981)	0.270 (0.178–0.361)	5.801	< 0.0001
WP	0.610 (0.433–0.787)	0.148 (0.105–0.191)	6.774	< 0.0001
CRP				
BP	0.013 (− 0.019–0.045)	0.033 (− 0.048–0.113)	0.798	0.425
WP	0.046 (0.033–0.059)	0.127 (0.091–0.162)	7.017	< 0.0001
ESR				
BP	0.008 (− 0.009–0.025)	0.037 (− 0.042–0.115)	0.92	0.358
WP	0.049 (0.037–0.060)	0.142 (0.109–0.175)	8.506	< 0.0001
SJC				
BP	0.024 (− 0.173–0.220)	0.009 (− 0.068–0.086)	0.235	0.814
WP	0.209 (0.146–0.272)	0.104 (0.073–0.135)	6.546	< 0.0001
TJC				
BP	0.372 (0.251–0.493)	0.217 (0.147–0.288)	6.064	< 0.0001
WP	0.173 (0.127–0.219)	0.118 (0.086–0.149)	7.341	< 0.0001
VAS-GH				
BP	0.074 (0.064–0.085)	0.515 (0.443–0.588)	13.934	< 0.0001
WP	0.029 (0.023–0.035)	0.167 (0.130–0.203)	8.948	< 0.0001

*DAS28-ESR* disease activity score in 28 joint including the erythrocyte sedimentation rate; *BP* between-person association; *WP* within-person association; *CRP* C-reactive protein; *ESR* erythrocyte sedimentation rate; *VAS-GH* patient-reported assessment of General Health on a 100-mm visual analog scale

**Fig. 2:**
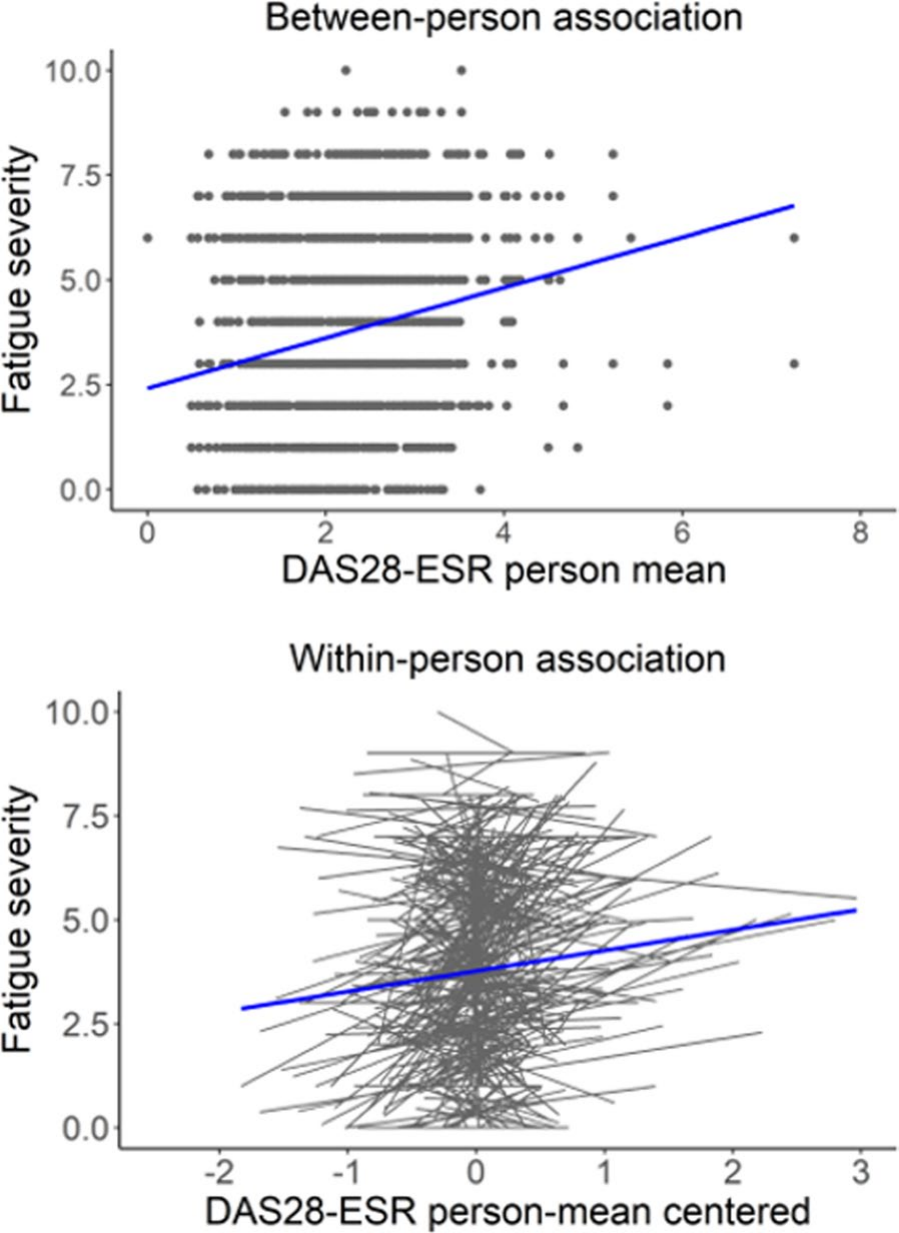
Between-person (upper panel) and within-person (lower panel) associations between disease activity and fatigue severity. *DAS28-ESR* disease activity score in 28 joints including the erythrocyte sedimentation rate

Both types of association were, however, only weak in magnitude. The low overall within-person association is illustrated in Fig. 2 (lower panel), showing a large variability in individual regression lines. Many patients showed strong positive associations between changes in disease activity and fatigue over time, but many patients also demonstrated no or even negative associations.

**Fig. 3:**
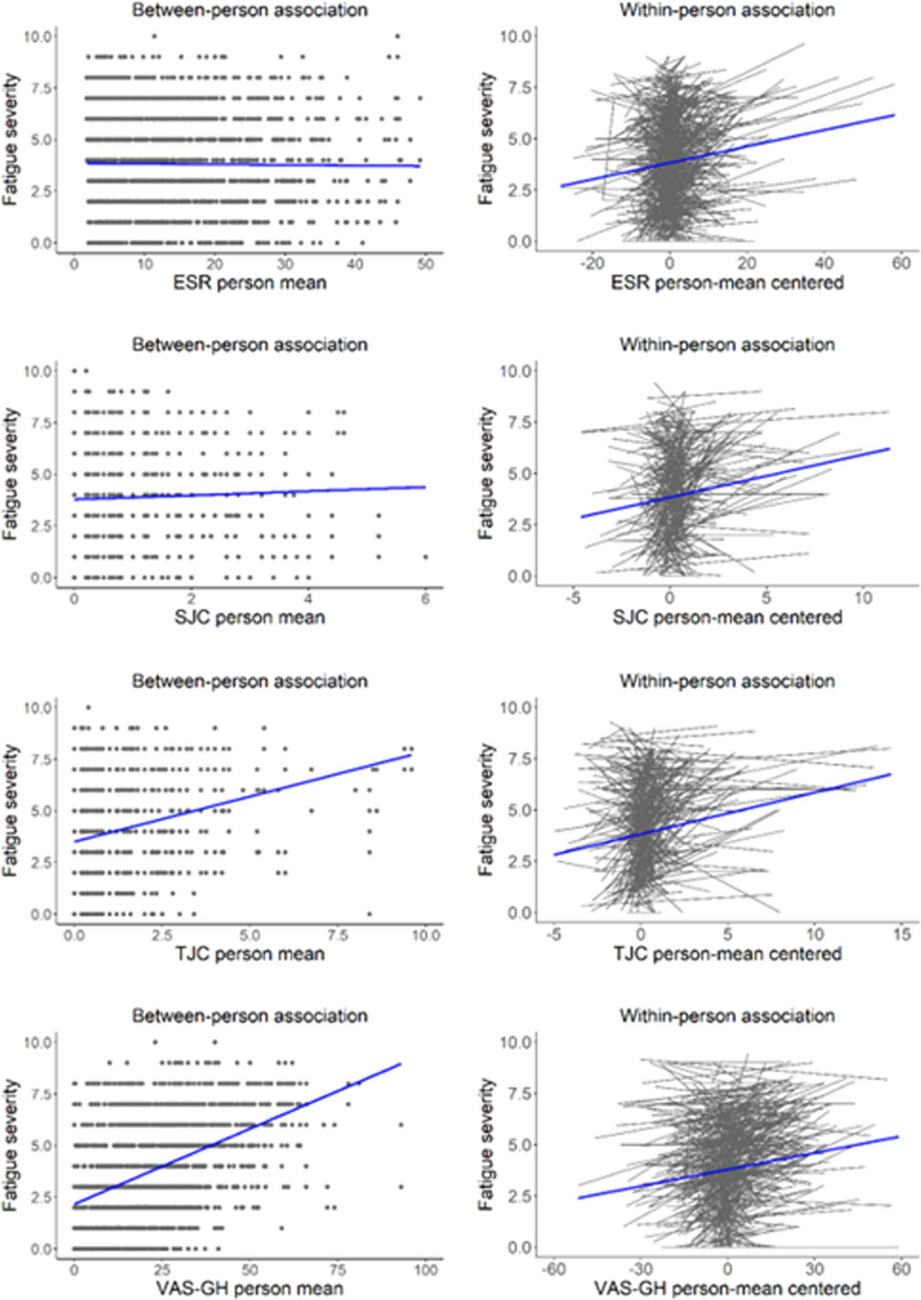
Between-person (left panel) and within-person (right panel) associations between individuals disease activity parameters and fatigue. *ESR* erythrocyte sedimentation rate; *SJC* swollen joint count; *TJC* tender joint count; *VAS-GH* patient reported assessment of General Health on a 100 mm visual analog scale

For the individual parameters of disease activity, a consistent difference between the objective and subjective parameters emerged (Table 3). Objective indicators of disease activity (CRP, ESR and SJC) were associated weakly within patients over time (β’s: 0.104–0.142, *p*’s < 0.0001), but not between patients. This indicates that changes in these markers in individual patients were associated with increased fatigue, but that patients with on average higher disease activity than other patients did not report more fatigue over time. In contrast, the TJC and VAS-GH were significantly associated both within and between patients, but with substantially stronger associations at the between-patient level. Especially the VAS-GH was strongly associated with fatigue between patients (β = 0.515, *p* < 0.0001) but only weakly within patients (β = 0.167, *p* < 0.0001). As with the composite DAS28-ESR scores, substantial individual differences were in the associations between separate disease activity parameters and fatigue (Fig. 3).

Overall, the segregated analyses confirmed that DAS28-ESR scores and fatigue are weakly associated both between- and within patients over time, but that these associations are quite different for both different patients and for different components of disease activity.

## Discussion

In this study, we demonstrated that in RA patients disease activity is related to their fatigue. However, this relation is highly individual and may also be absent of negative in some patients. In addition, the relationship is different for the individual components of disease activity. Associations between subjective measures of disease activity and fatigue mostly reflect more stable between-patient differences, whereas objective markers of inflammation show significant positive associations only within patients.

Biomedically, a positive association between fatigue and disease activity can be explained by activity of the inflammatory process that comes with acute phase reactions and stimulation of cytokines and inflammatory cells. Also, fatigue in active RA can be the understandable consequence of the increased energy requirement in physical activities due to painful and swollen joints combined with muscle weakness [35]. From this pathophysiology, fatigue in RA patients with high disease activity can be reduced by successful suppression of inflammation. However, persistent fatigue in some RA patients with low levels of objective disease activity seems to suggest a mechanism independent from inflammation. It is hypothesized that several behavioral, psychological and cognitive mechanisms drive this process in a complex way [36]. This is reflected in a biopsychosocial model in which it is assumed that factors are to be involved to varying degrees in individual patients and that these factors influence each other in diverse ways in different patients. Physical functioning, poor mental status, sleep disturbance, pain, depression and anxiety have often been found to be independent variables associated with fatigue in multivariate analyses [37]. Hewlett et al. also proposed a dynamic model with bidirectional interactions between disease processes, cognitive and behavioural factors and personal life issues and assumed the influence of this factors to vary between individuals and within individuals at different times [38].

The use of different (composite) indicators of disease activity and analyses, which do not distinguish between associations between persons and within persons, may have led to the conflicting results in earlier research. The results of the analysis used in our study, which separate between-patients and within-patients associations, may explain why these previous studies generally found either weaker or contradictory associations between disease activity and fatigue. In our study, we included the results of the aggregated and disaggregated analysis to show what happens when within-person and between-person associations are statistically separated. The results confirm the varying relationship between different disease activity measures and fatigue both between and within individuals. Our study shows a weak significant association between fatigue and the objective components of disease activity, e.g., inflammation, within individuals and even no significant association of these factors between individuals. Additionally, we have clearly illustrated that the within person association between inflammation and fatigue varies widely between individual patients from strong and positive to absent or even negative.

The stronger association between fatigue and the subjective components of disease activity, e.g., tender joints and general health, between persons may, although not directly inferred from the current study, support the idea that fatigue may be a more common symptom of chronic disease rather than a disease-specific symptom. In particular, patients’ perceived general health will to some extent depend on factors that are not always disease specific. Menting et al., already demonstrated that variance in fatigue severity in different chronic diseases, including RA, can largely be explained by non-disease-specific factors like sex, age, motivational and concentration problems, pain, sleep disturbance, physical functioning, reduced activity and lower self-efficacy [39]. They therefore argued for a transdiagnostic approach that focuses on the individual patient’s needs.

Given the various disease-specific and non-disease-specific factors that influence fatigue, the approach to this important patient reported symptom will have to be multidimensional. Proper multidimensional management of fatigue starts by making it an important topic for discussion during the consultation between patient and healthcare professional. Although many patients report fatigue as a major symptom, it is not structurally communicated at the rheumatology outpatient clinic. It has been demonstrated that fatigue was communicated in only 6% of the total consultation time and that in most cases the patient initiated the communication on fatigue mostly using cues to express their worries instead of communicating their concerns directly [40]. As a result, healthcare professionals will have an important role in communicating about fatigue and recognizing concerns that are indirectly reported by patients.

Our study was subjected to some strengths and weaknesses [25]. The cohort used for this study was one of the largest studies in real life, where prospectively and stringently, disease activity as well as fatigue was measured after stopping one of the most successful anti rheumatic therapies, i.e., TNFi, while being in remission. Consequently, flares with changes in fatigue and disease activity could be expected. Our statistical approach proved suitable to disentangle and illustrate variation between two or more variables in different individuals, providing a more detailed insight than standard cross-sectional and longitudinal analyses. One of the limitations of our study is that flares in the POEET cohort where immediately followed by restart of TNFi, so major and long-lasting flares are rare. However, the data as they are with mostly mild and short flares clearly illustrated the overall and individual associations between the different indices of disease activity and fatigue. Major and long-lasting flares would likely only have strengthened this conclusion.

Our study was explorative in design as a first demonstration that the relation between disease activity and fatigue is different in individual patients. Future research may focus on the possible reasons for these differences and on identifying groups of patients with the same or absent associations. Now that most patients with RA are able to achieve remission, fatigue remains one of the most debilitating symptom for some patients. We believe, that our data support the need for a more personalized approach to further improve the management of fatigue in RA. Optimal anti-inflammatory treatment may need to be combined with neuro-psychological interventions based upon the needs of individual RA-patients. Many personal factors, which healthcare providers may not always know or be able to influence, will contribute to varying degrees to fatigue in RA patients. Differences in associations between disease activity and fatigue between different patients must be first recognized before knowledge and interventions in this area can be improved. RA patients suffering from fatigue sometimes, but not always, have high disease activity and therefore do not always need more or different medication. But they will all need to be approached and advised with respect and to the best of our knowledge.

## Conclusion

Our results show that in some patients inflammatory disease activity and fatigue are related, while in other patients this association seems absent. More research on the complex relation between disease activity and fatigue in RA and other inflammation driven diseases is necessary, but our results point to the need for a more individually targeted approach of fatigue in RA, both in research and daily clinical practice.

## Data Availability

The datasets supporting the conclusions of this article are not publicly available due to legal and ethical reasons but are available from the corresponding author on reasonable request.

## Abbreviations

RA: Rheumatoid arthritis
DAS-28: Disease activity score in 28 joints
TNFi: Tumor Necrosis Factor inhibitor
ESR: Erythrocyte sedimentation rate
CRP: C-reactive protein
TJC: Tender joint count
SJC: Swollen joint count
VAS-GH: Patient-reported assessment of general health on a 100-mm visual analog scale
DAS28-ESR: Disease activity score in 28 joints including the erythrocyte sedimentation rate
BRAF-NRS: Bristol Rheumatoid Arthritis Fatigue-Numerical Rating Scale
RF: Rheumatoid factor
Anti-CCP: Anti-cyclic citrullinated peptide
